# An Experimental Program for Effective Communication of Middle-Aged and Older Adults With Pharmacists in Taiwan

**DOI:** 10.1093/geroni/igaa057.1597

**Published:** 2020-12-16

**Authors:** Yi-Wei Sun, Hui-Ching Weng

**Affiliations:** 1 National Cheng Kung University, Taichung, Taiwan (Republic of China); 2 National Cheng Kung University, Tainan City, Tainan, Taiwan (Republic of China)

## Abstract

Plenty of potential drug-related problems was noted in the middle-aged and older adults. Effective communication is essential for pharmacists to coordinate their opinions with patients to improve the effectiveness of drug treatment and reduce drug-related problems. However, few studies focused on patients’ own abilities to communicate with pharmacists even though it is vital for the effectiveness of medical treatment. The purpose of this study was to design a communication training program for middle-age and older adults not only to enhance their understanding of pharmacists’ responsibilities and the importance to communicate with pharmacists, but also to improve their self-efficacy about drug-related communication and the ability to list their latest medication. A non-randomized control-group pretest-posttest design was conducted in Tainan City. Through individual interviews of five pharmacists and a focus group interview, we ensured the appropriateness and expertise of the training program. 179 adults over 55 years old were distributed into experimental group or control group. The experimental group underwent the training program after the pretest, while no intervention was provided to the control group. Face-to-face and role-playing sessions were in process with a training booklet in the courses. Both groups completed the posttest four weeks after the pretest. This communication training program appears to be effective since the knowledge of pharmacists’ responsibilities and self-efficacy of drug-related communication were significantly improved in the experimental group (p<.05) rather than control group. Completion of the recent medication list was also improved from 40.3% to 80.6% and was significantly higher than the control group.

